# Supplementary material for: Splice-Junction-Based Mapping of Alternative Isoforms in the Human Proteome
Source: Cell Rep. Author manuscript; Available in PMC 2020 Jan 15. (PMC6961840; doi:10.1016/j.celrep.2019.11.026)
Supplement: 3 [file NIHMS1546469-supplement-3.zip › DF2/PXD006675/LeftVentricle-131-Q92736-VLDIANVLFHLEQVEHPQR.pdf]

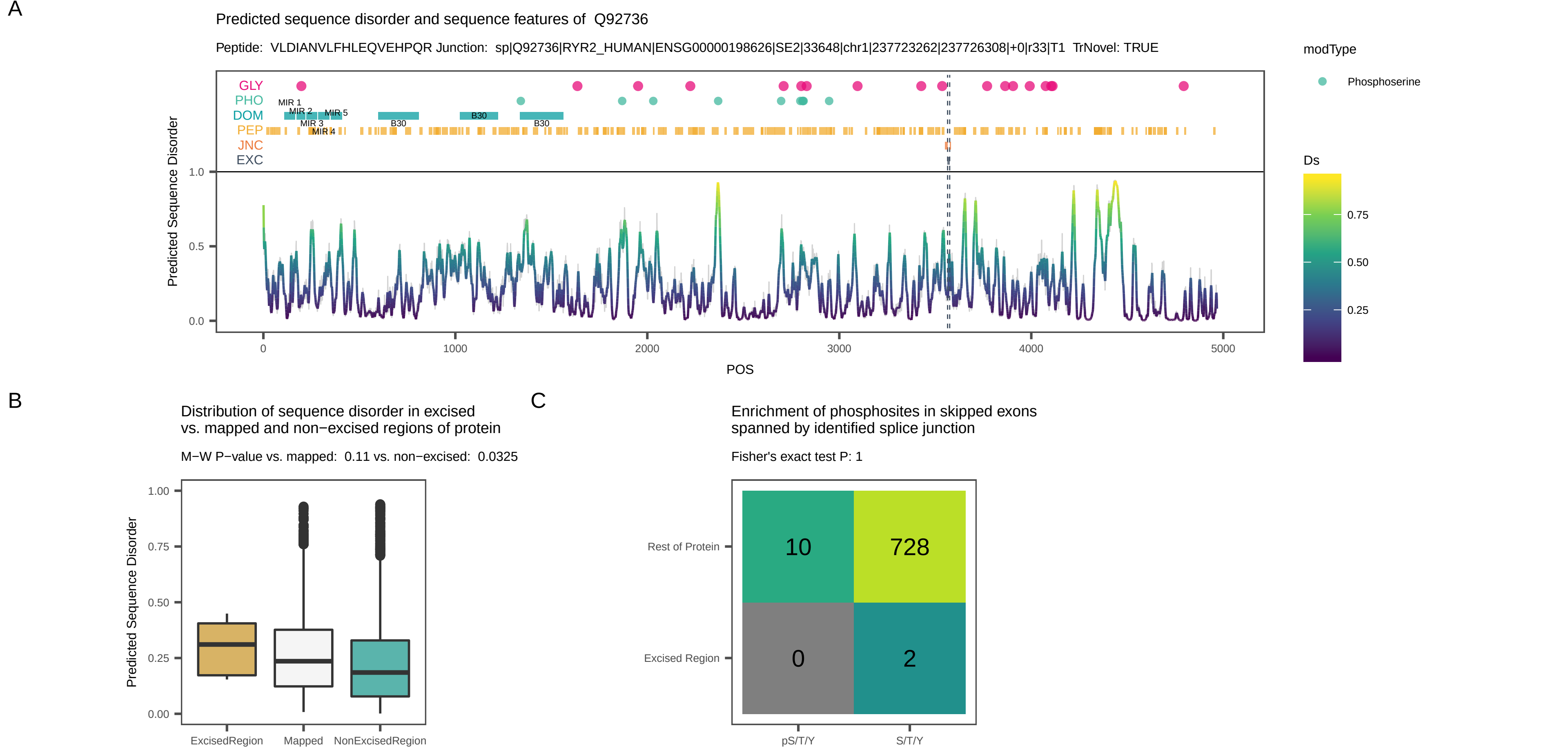
A
Predicted sequence disorder and sequence features of Q92736
Peptide: VLDIANVLFHLEQVEHPQR Junction: sp|Q92736|RYR2_HUMAN|ENSG00000198626|SE2|33648|chr1|237723262|237726308|+0|r33|T1 TrNovel: TRUE
GLY
PHO
DOM
PEP
JNC
EXC
MIR 1
MIR 2
MIR 3
MIR 4
MIR 5
B30
Predicted Sequence Disorder
POS
modType
Phosphoserine
Ds
0.75
0.50
0.25
B
Distribution of sequence disorder in excised vs. mapped and non−excised regions of protein
M−W P−value vs. mapped: 0.11 vs. non−excised: 0.0325
Predicted Sequence Disorder
ExcisedRegion
Mapped
NonExcisedRegion
C
Enrichment of phosphosites in skipped exons spanned by identified splice junction
Fisher's exact test P: 1
Rest of Protein
Excised Region
10
728
0
2
pS/T/Y
S/T/Y
